# Risk factors for unplanned return to the operating room within 24 hours: A 9-year single-center observational study

**DOI:** 10.1097/MD.0000000000028053

**Published:** 2021-12-10

**Authors:** Feng-Chen Kao, Yun-Chi Chang, Tzu-Shan Chen, Ping-Hsin Liu, Yuan-Kun Tu

**Affiliations:** a Department of Orthopedics, E-Da Hospital, Kaohsiung, Taiwan; b School of Medicine for International Students, I-Shou University, Kaohsiung, Taiwan; c Department of Anesthesia, E-Da Hospital, Kaohsiung, Taiwan; d Department of Medical Research, E-Da Hospital, Kaohsiung, Taiwan.

**Keywords:** bleeding, operation room, return, risk factor, unplanned

## Abstract

The purpose of the retrospective case–control study was to identify the causes of and risk factors for unplanned return to the operating room (uROR) within 24 hours in surgical patients.

We examined 275 cases of 24-hour uROR in our hospital from January 2010 to December 2018. The reasons for 24-hour uROR were classified into several categories. Controls were randomly matched to cases in a 1:1 ratio with the selection criteria set for the same surgeon and operation code in the same corresponding year.

The mortality rate was significantly higher in patients with 24-hour uROR (11.63% vs 5.23%). Bleeding was the most common etiology (172/275; 62.55%) and technical error (14.5%) also contributed to 24-hour uROR. The clinical factors that led to bleeding included a history of liver disease (*P* = .032), smoking (*P* = .002), low platelet count in preoperative screening (*P* = .012), and preoperative administration of antiplatelet or anticoagulant agents (*P* = .014).

Clinicians should recognize the risk factors for bleeding and minimize errors to avoid the increase in patient morbidity and mortality that is associated with 24-hour uROR.

**Level of Evidence:** Level IV.

## 1 Introduction

Return to the operating room (ROR) is a clinically discrete event that is difficult to quantify. Determining the clinical indications for ROR is often complicated.^[1]^ ROR may occur because of a planned, staged procedure for patients with complex clinical conditions.^[2]^ Unplanned ROR (uROR) reflects indicates the quality of surgical outcomes.^[3–7]^ This event attracts more attention in surgical care than does ROR because it occurs more frequently^[6]^; is an easily identified discrete event; and is associated with increased morbidity (both short and long term),^[4,7]^ longer hospital stay, and greater resource utilization and associated costs.^[6,8–11]^

The evaluation of uROR requires reliable and valid measurements, timely data collection, and the cooperation within the care teams.^[1]^ Reported uROR rates vary widely from 0.6% to 9%,^[12,13]^ consistent with the rate documented in the database of the American College of Surgeons National Surgical Quality Improvement Program.^[14]^ The following factors are associated with a higher uROR rate: surgical type,^[4,9]^ surgical acuity,^[15]^ surgical technique, patient comorbidities,^[4,10,16]^ and differences in coding practices between institutions.^[17]^ Studies on ROR have mainly focused on total ROR, uROR, or 30-day ROR.^[1,2,12–14]^ To the best of our knowledge, none have examined 24-hour uROR. The need to perform a second operation within 24 hours of the first operation implies a serious clinical condition. This is often alarming for both the patient and surgeon and carries a relatively high risk of morbidity and mortality.

We conducted a study using the data collected from our institution, a tertiary hospital with a high surgical volume, to identify the causes of and risk factors for 24-hour uROR. Recognition of such risk factors may facilitate the identification of patients who would benefit from early treatment to prevent 24-hour uROR. It may also remind clinical physicians to make the necessary arrangements when encountering similar clinical situations.

## 2 Methods

### 2.1 Study structure and characteristics

We conducted a retrospective observational case–control study using medical records maintained by the operating room committee of our hospital. The study was reported according to STROBE guidelines.^[18]^ No individual was clinically involved in this study. We retrospectively reviewed the medical records of all cases. The level-A Institutional Review Board of E-Da Hospital approved the study protocol and exempted the investigation from a full review.

Our institution is a tertiary hospital with a high monthly surgical volume of approximately 2500. These procedures are performed under moderate-to-deep anesthesia. Our database provides data on patients undergoing general, colorectal, pediatric, cardiovascular, thoracic, oral, plastic, gynecologic, urologic, otolaryngologic, orthopedic, and neurological surgery.

Herein, we included patients who made an uROR within 24 hours of an operation at our hospital from January 2010 to December 2018. Controls were randomly matched to cases in a 1:1 ratio. The risk factors for 24-hour uROR were identified. After study completion, the results were assessed by our hospital network.

### 2.2 Study data, study groups, and outcomes

Since 2010, all cases of 24-hour uROR at our hospital have been coded every month by our institution's operating room committee. A case of 24-hour uROR is coded based on the following criteria: uROR within 24 hours of the index operation, uROR being likely related to the previous operation, and no indication or documentation (including operative notes or patient charts) of any planned or staged surgery before or during the index operation.^[19]^

**Figure 1:**
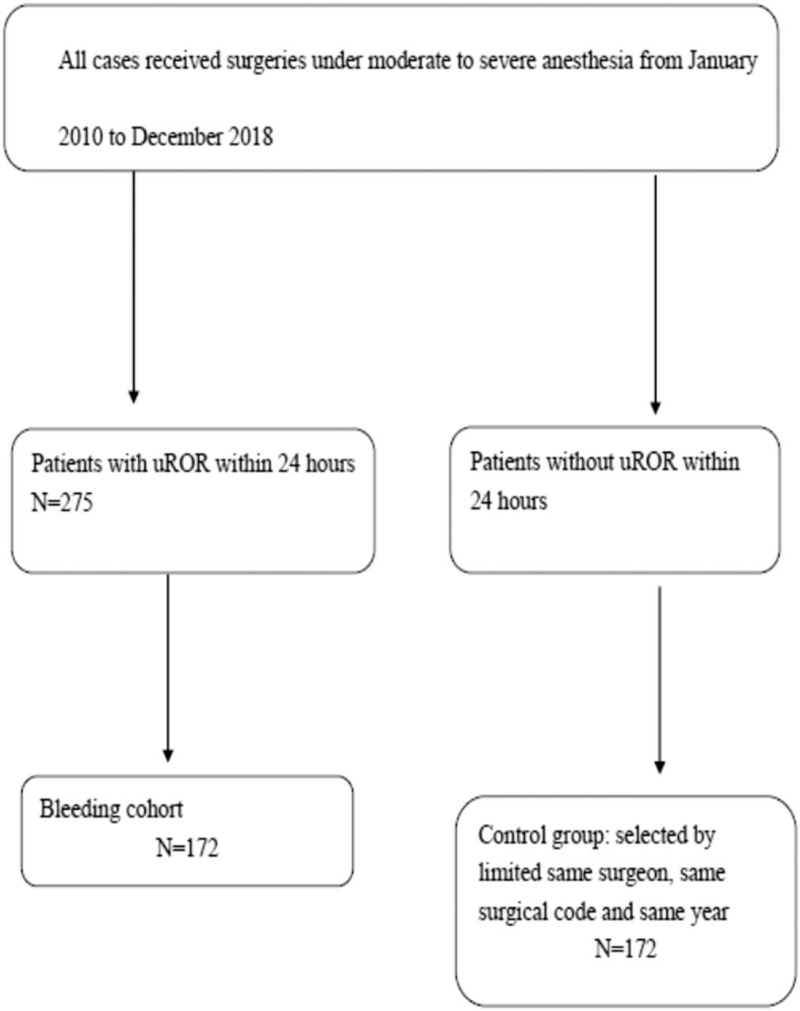
Study flowchart. uROR = unplanned return to the operating room.

At our institution, cases of 24-hour uROR are identified from the monthly recorded dataset. This information is sent to the surgeon and corresponding supervisor, who are required to explain the reasons for 24-hour uROR and confirm that the case was not planned or miscoded. Final verification of 24-hour uROR cases is conducted by the chief of the operating room committee according to all this documentation and responses from surgeons and supervisors. The study flowchart is presented in Figure 1.

The reasons for 24-hour uROR were classified into the following categories: bleeding, vascular obstruction, endotracheal extubation, endotracheal compression, increased intracranial pressure, hydrocephalus, and other. We selected the most common reason for 24-hour uROR as the research target to focus on the relatively urgent and potentially correctable factors. Moreover, we assessed whether the uROR was due to technical error by reviewing the documentation and surgeon and supervisor responses.

Controls that did not have 24-hour uROR or any uROR were randomly matched to cases in a 1:1 ratio, with the selection criteria set for the same surgeon for the same operation code in the same corresponding year. We collected data on patient characteristics, including smoking habits, alcohol use, and comorbidities, average hospital stay, surgical department, operative variables, and the operation conducted as an emergency procedure. We also analyzed the impact on the final mortality of factors such as 24-hour uROR, gender, body mass index, smoking, drinking, betel nut chewing, baseline comorbidities, and duration of anesthesia.

### 2.3 Statistical analysis

We employed the *t* test for analyzing continuous variables and the chi-square test for analyzing categorical variables. Multiple logistic regression was performed. All analyses were conducted using Statistical Package for the Social Sciences (SPSS) version 24.0 software (IBM Corporation, Armonk, NY, USA). A *P* value of <.05 was considered statistically significant.

## 3 Results

### 3.1 Rate of and reasons for 24-hour uROR

Of a total of 292,500 surgeries performed under moderate-to-deep anesthesia at our hospital from January 2010 to December 2018, 275 (0.13%) cases of 24-hour uROR were identified. In total, 33 patients died after the uROR; the mortality rate was 12%.

**Figure 2:**
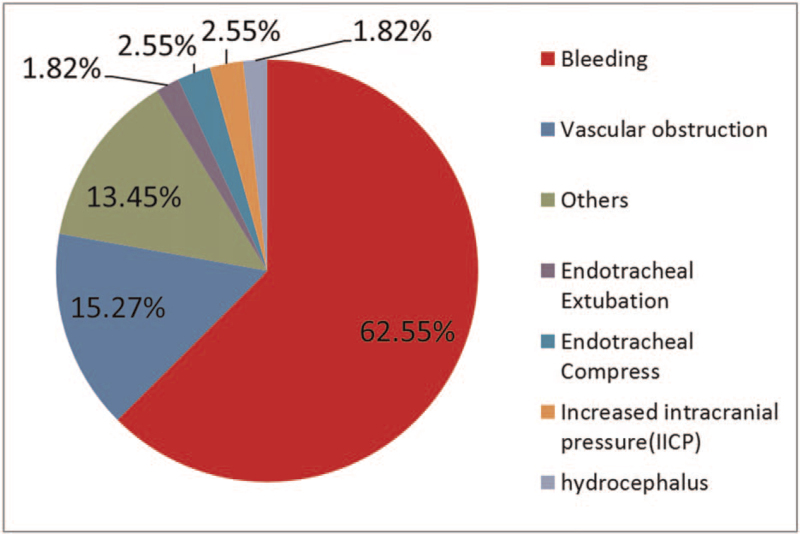
Potential causes of unplanned return to the operating room (uROR) within 24 hours.

Figure 2 presents the distribution of the reasons for 24-hour uROR. Among them, bleeding was the most common etiology (172/275; 62.55%), followed by vascular obstruction (15.27%) and others (13.45%). Cases with more than 1 uROR involved cardiac surgeries, urologic surgeries, chest surgeries, neurologic surgeries, and plastic surgeries. The detailed surgical procedures are listed in Table 1. Furthermore, 40 (14.5%) cases were due to technical error.

**Table 1:** surgical procedures and departments in patients with more than one time uROR.

Division	Surgical procedures	n	uROR rate
Cardiac surgeries	Single valve replacement	8	4.26%
	Coronary artery bypass surgery (CABG)	5	2.66%
Urologic surgeries	Transurethral resection of the prostate(TURP)15–50 gm	7	3.72%
	Thulium laser enucleation of prostate in BPH	5	2.66%
Chest surgeries	Thoracoscopic lobectomy	3	1.60%
Neurologic surgeries	Removal of Intracerebral of hematoma	13	6.91%
	Transphenoidal removal of pituitary adenoma	6	3.19%
Plastic surgeries	Microvascular free flap	6	3.19%
	Local flap	5	2.66%

### 3.2 Risk factors for bleeding

We matched the 172 cases with bleeding as the cause of 24-hour uROR with controls in a 1:1 ratio. The baseline characteristics of both cases and controls are listed in Table 2.

**Table 2:** Characteristics and primary outcomes of patients with/without unplanned return to operation room (uROR) within 24 hours caused by bleeding.

	Nonreoperations (n = 172)	Unplanned reoperations (n = 172)	*P*
Is/not an emergent surgery			.505
Emergency surgery	33 (19.19%)	38 (22.09%)	
Elective surgery	139 (80.81%)	134 (77.91%)	
ASA			.634
1	13 (7.56%)	13 (7.56%)	
2	65 (37.79%)	66 (38.37%)	
3	78 (45.35%)	70 (40.70%)	
4	16 (9.30%)	22 (12.79%)	
5	0 (0.00%)	1 (0.58%)	
Operative time			.134
<2 h	65 (37.79%)	45 (26.16%)	
2–4 h	51 (29.65%)	66 (38.37%)	
4–6 h	29 (16.86%)	31 (18.02%)	
Over 6 h	27 (15.70%)	30 (17.44%)	
Length of stays	11.42 ± 10.94	18.53 ± 20.81	<.0001
Mortality			.033^∗^
Yes	9 (5.23%)	20 (11.63%)	
No	163 (94.77%)	152 (88.37%)	
Gander		.313	
Male	105 (38.95%)	114 (33.72%)	
Female	67 (61.05%)	58 (66.28%)	
Age	58.424 ± 17.02	57.60 ± 15.45	.638
Age group			.500
≤44 yr	37 (21.51%)	33 (19.19%)	
45–59 yr	45 (26.16%)	57 (33.14%)	
60–74 yr	59 (34.30%)	55 (31.98%)	
≥75 yr	31 (18.02%)	27 (15.70%)	
BMI kg/m^2^			.726
<25 (normal)	88 (51.16%)	82 (47.67%)	
25–29 (overweight)	56 (32.56%)	63 (36.63%)	
≥30 (obese)	28 (16.28%)	27 (15.70%)	
History of hypertension			.279
Yes	74 (43.02%)	84 (48.84%)	
No	98 (52.7%)	88 (47.3%)	
History of heart disease			.776
Yes	29 (16.86%)	31 (18.02%)	
No	143 (83.14%)	141 (81.98%)	
History of myocardial infarct			.557
Yes	7 (4.07%)	5 (2.91%)	
No	165 (95.93%)	167 (97.09%)	
History of congestive heart failure			.829
Yes	11 (6.40%)	12 (6.98%)	
No	161 (93.60%)	160 (93.02%)	
History of diabetes			.471
Yes	45 (26.16%)	51 (29.65%)	
No	127 (73.84%)	121 (70.35%)	
History of cerebrovascular disease			.161
Yes	10 (5.81%)	17 (9.88%)	
No	162 (94.19%)	155 (90.12%)	
History of chronic pulmonary disease			.125
Yes	8 (4.65%)	3 (1.74%)	
No	164 (95.35%)	169 (98.26%)	
History of ulcer disease			.085
Yes	24 (13.95%)	14 (8.14%)	
No	148 (86.05%)	158 (91.86%)	
History of mild liver disease			.032^∗^
Yes	1 (0.58%)	7 (4.07%)	
No	171 (99.42%)	165 (95.93%)	
Moderate or severe renal disease			.560
Yes	13 (7.56%)	16 (9.30%)	
No	159 (92.44%)	156 (90.70%)	
Cancer			.718
Yes	49 (71.51%)	46 (73.26%)	
No	123 (28.49%)	126 (26.74%)	
Current smoker			.002^∗∗^
Yes	33 (19.19%)	59 (34.30%)	
No	139 (80.81%)	113 (65.70%)	
Alcohol			.756
Yes	23 (13.37%)	25 (14.53%)	
No	149 (86.63%)	147 (85.47%)	
Chewing betel nut			1.000
Yes	159 (92.44%)	159 (92.44%)	
No	13 (7.56%)	13 (7.56%)	
Received antiplatelate drug and anticoagulant agents before operation			.014^∗^
Yes	14 (8.14%)	29 (16.86%)	
No	158 (91.86%)	143 (83.14%)	
Low platelate count in preoperation screening			.012^∗^
Yes	27 (15.70%)	46 (26.74%)	
No	145 (84.30%)	126 (73.26%)	

The mortality rate in the case group after the 24-hour uROR was significantly higher than that in the control group (11.63% vs 5.23%). The average hospital stay in the case group after the 24-hour uROR was also significantly longer than that in the control group (18.53 ± 20.81 days vs 11.42 ± 10.94 days).

Bleeding occurrence was not affected by surgical department, surgical duration, level of anesthesia, or the emergency status of the operation. Clinical factors that potentially led to bleeding included a history of liver disease (*P* = .032), smoking (*P* = .002), low preoperative platelet count (*P* = .012), and preoperative administration of antiplatelet or anticoagulant agents (*P* = .014). The results are presented in Table 1.

### 3.3 Risk factors for mortality

Multiple logistic regression revealed a significantly higher incidence of mortality in the 24-hour uROR group than in the control group (crude hazard ratio [HR], 2.383; 95% confidence interval, 1.053–5.395; *P* = .037). However, this difference was not significant after adjustment for 24-hour uROR, sex, body mass index, smoking, drinking, betel nut chewing, baseline comorbidities, and duration of anesthesia (adjusted HR, 2.11; *P* = .095; Table 3).

**Table 3:** Risk factors of mortality analyzed by multiple variable logistic regression test.

		Crude sHR (95% CI)	*P* value	Adjusted sHR (95% CI)	*P* value
Unplanned return	No	Ref		Ref	
	Yes	2.383 (1.053–5.395)	.037	2.11 (0.878–5.078)	.095
Operative time		1.027 (0.933–1.131)	.582	1.032 (0.912–1.167)	.618
Gender	Female	Ref		Ref	
	Male	1.883 (0.780–4.541)	.159	2.173 (0.802–5.887)	.127
BMI kg/m^2^		0.947 (0.868–1.033)	.22	0.925 (0.834–1.026)	.139
History of hypertension		1.108 (0.518–2.373)	.791	1.01 (0.404–2.520)	.986
History of heart disease		1.927 (0.810–4.585)	.138	1.460 (0.377–5.649)	.583
History of myocardial infarct		0.987 (0.123–7.927)	.99	0.490 (0.043–5.625)	.567
History of congestive heart failure		2.493 (0.787–7.895)	.12	1.452 (0.273–7.720)	.662
History of diabetes		1.179 (0.517–2.69)	.695	1.42 (0.241–8.375)	.697
History of cerebrovascular disease		0.859 (0.193–3.825)	.842	0.792 (0.161–3.902)	.774
History of chronic pulmonary disease		1.089 (0.134–8.822)	.936	1.370 (0.211–26.872)	.792
History of ulcer disease		0.268 (0.035–2.031)	.203	0.185 (0.018–1.870)	.153
History of mild liver disease		1.571 (0.187–13.233)	.678	2.661 (0.238–29.737)	.427
Moderate or severe renal disease		1.283 (0.364–4.525)	.699	1.251 (0.298–5.245)	.760
Cancer		0.394 (0.133–1.164)	.092	0.296 (0.081–1.084)	.066

## 4 Discussion

uROR has a high rate of severe adverse events (48%–79%).^[20–22]^ Analyzing the corresponding data on complications requiring uROR in all surgical specialties may improve treatment quality, risk management, and quality of care.^[6,23,24]^ Lin et al^[3]^ reported that patients with 30-day uROR had more preoperative comorbidities and underwent longer and more complex operations. Occasionally, the patients experienced more than one uROR during their hospitalization, resulting in the extension of their hospital stays and higher rates of postoperative mortality and morbidity after the second operation.^[3]^ Herein, the mortality rate in the 24-hour uROR group was higher than that in the control group (crude HR, 2.383; *P* = .037 and adjusted HR, 2.11; *P* = .095; Table 3). We centered our investigation on the relatively dangerous situation in which patients underwent reoperation within 24 hours. Overall, 24-hour uROR led to longer hospital stays and higher mortality rates (12%). Therefore, minimizing the risk of 24-hour uROR is crucial to improving the quality of surgical care.

Lawson reported that approximately 3% of all surgical procedures are accompanied by some degree of bleeding.^[25]^ Various factors can lead to uROR within 24 hours. Lin et al^[3]^ reported that surgical site infection and hemorrhage are the most common reasons for 30-day uROR. In our study, the most common reason for 24-hour uROR was bleeding (62.55%). Thus, identifying and preventing the causes of surgery related bleeding is warranted.

Some clinical conditions or drugs, such as thrombocytopenia, platelet disorders, mild-to-moderate inherited coagulation defects, and oral anticoagulant and platelet-inhibiting drug administration, may be associated with a higher risk of bleeding in patients undergoing major surgery.^[25–27]^ The majority of bleeding events in cases of uROR have been reported to be induced by the administration of postoperative nonsteroidal anti-inflammatory drugs or anticoagulants within 24 hours of the index operation. Other studies have indicated that some structural or technical defects during surgery, such as an undone ligature, failed clamp, or partially secured vessel, may lead to bleeding problems. Furthermore, an excessively long surgical duration may contribute to such occurrences.^[25,28]^ In addition, concomitant medical conditions (eg, hepatic or renal dysfunction) and herbal medications may have deleterious effects on hemostatic tendencies. For example, the consumption of herbal products induces liver injury in patients with obesity undergoing weight reduction programs.^[27,29]^ In our study, factors associated with bleeding events in patients experiencing 24-hour uROR were a history of liver disease, smoking habits, low preoperative platelet count, and the preoperative administration of antiplatelet or anticoagulant agents.

Coagulopathies may be caused by multiple factors, such as physiological disturbances, hemostatic dysfunction, and plasma abnormalities.^[30]^ The diagnosis and management of perioperative coagulopathies remain challenging.^[30]^ Allogeneic blood transfusion may be necessary for managing perioperative hemostatic dysfunction. Identifying patients with an increased bleeding risk before surgery through routine preoperative screening of platelet count, prothrombin time, and partial thromboplastin time is pertinent for optimal surgical outcomes. However, determining the appropriate time at which to read those laboratory results and make adequate preoperative arrangements to correct hemostatic abnormalities remains challenging. During emergency surgery, surgeons may not have the time to focus on correcting hemostatic dysfunction. However, we did not detect a significant difference in bleeding occurrence between emergency and elective surgeries.

Technical error can also lead to 24-hour uROR. Herein, 14.5% of such technical error, as determined by examining feedback from surgeons and their supervisors. To prevent technical error from occurring, surgeons must undergo continual training and a prompt and appropriate feedback system for surgical performance must be established.

Our study has some limitations. This was a retrospective study in which data collected over 9 years were considered. Health care, surgical instruments and techniques, and the experience of the surgeons in question and surgical techniques have all evolved overinevitably evolved over this period; this may have resulted in bias. Moreover, the risk factor for 24-hour uROR could not be reflected immediately to clinical physicians. However, we identified some risk factors that are potentially correctable through changes in clinical treatment protocols and knowledge sharing to reduce the rate of 24-hour uROR at our institute.

In conclusion, bleeding was the most common cause of 24-hour uROR, and technical error was a contributor to 24-hour uROR. Clinicians should recognize the risk factors for bleeding and minimize technical error to avoid the increase in patient morbidity and mortality that is associated with 24-hour uROR.

## Acknowledgments

We thank Wallace Academic Editing for editing this manuscript.

## Author contributions

**Conceptualization:** Ping-Hsin Liu, Yuan-Kun Tu.

**Data curation:** Yun-Chi Chang.

**Formal analysis:** Tzu-Shan Chen.

**Writing – original draft:** FengChen Kao.
